# Effects of quarantine and vaccination on the transmission of Lumpy skin disease: A fractional approach

**DOI:** 10.1371/journal.pone.0327673

**Published:** 2025-07-17

**Authors:** Nada A. Almuallem, Ram Pratap Chauhan

**Affiliations:** 1 Department of Mathematics and Statistics, Faculty of Science, University of Jeddah, Jeddah, Saudi Arabia; 2 Department of Mathematics, Amrita Vishwa Vidyapeetham, Amaravati, Andhra Pradesh, India; Central Laboratory for Evaluation of Veterinary Biologics, Agricultural Research Center, EGYPT

## Abstract

Lumpy skin disease (LSD) is a viral infection that affects cattle, resulting from the lumpy skin disease virus. This study investigates the impact of vaccination and quarantine strategies on LSD outbreaks. This study analyzes a nonlinear model for LSD using the Caputo fractional operator. The positivity and boundedness of the model’s solutions are confirmed. Equilibrium points for both disease-free and endemic states are derived, and the basic reproduction number is determined using the next-generation matrix method. An equilibrium point stability analysis is performed. The bifurcation graphs for steady states are presented. Sensitivity indices are calculated to identify the parameters that most significantly influence the dynamics of the disease. The dynamical behavior is analyzed using a Lagrange polynomial interpolation-based numerical scheme. The results demonstrate that increasing the vaccination rate can lead to the elimination of the disease. Increasing movement restrictions for the exposed cattle population reduces infection rates, but does not eliminate the disease. In addition, the spread of LSD is more sensitive to the recruitment of susceptible individuals.

## 1 Introduction

Numerous diseases can affect cattle, with Lumpy skin disease being the most significant among them. LSD is a viral infection caused by Capripoxvirus. Symptoms in animals can vary depending on the way the disease progresses. However, common signs in cattle and other ruminants include distinct skin lumps and/or nodules, fever, and a loss of appetite [1,2]. It results in reduced milk supply, weight reduction, and, in the worst cases, mortality. LSD is primarily spread through vector-borne transmission, with mosquitoes, biting flies, and ticks acting as mechanical vectors for the virus among susceptible cattle [3]. While direct contact between sick and susceptible animals, through injuries to the skin or bodily fluids can facilitate transmission, this mode is less crucial in natural environments in the absence of insect vectors. Furthermore, indirect transmission via contaminated fomites, such as feed, water, and equipment, contributes to disease spread and highlights the necessity for rigorous biosecurity measures [4]. LSD significantly affects animal health, productivity, and economies, leading to trade restrictions, reduced income, and food shortages for affected farmers and communities [2]. Animals exposed to LSD typically show symptoms after an incubation period of around two to four weeks [5]. The disease was first identified in Zambia in 1929. South Africa faced an LSD outbreak in 1949, resulting in a severe economic crisis. Since the year 2000, LSD outbreaks have affected multiple countries across the Middle East. Furthermore, the disease has been documented in various European countries, including Greece, Bulgaria, Turkey, Kosovo, and Russia. Its spread has extended into Asia, with cases reported in India, Bangladesh, Myanmar, Pakistan, Nepal and Thailand [6]. While there is no cure, effective strategies to control the spread of the disease include vaccination, vector elimination, and the implementation of quarantine or isolation of cattle showing symptoms [7–9]. In [10] and [11], it was reported that restricting cattle movement, removing those with illness, quarantining them before reintegration to the herd, and preventing herd mixing during feeding and watering are effective strategies for infection prevention. Therefore, farmers, veterinarians and other stakeholders must work together to prevent the spread of LSD and minimize the negative impacts using various strategies.

Mathematical modeling has gained prominence across diverse fields as a powerful tool for analyzing and solving complex real-world problems. It facilitates the analysis of dynamic processes and supports more reliable predictions of future outcomes. One key application is in the study of infectious disease dynamics, which is crucial for developing effective treatments, control strategies, and preventive measures. Mathematical models have played a crucial role in understanding outbreaks such as measles [12], Ebola virus disease [13,14], HIV [15], Monkeypox infection [16], particularly in the context of LSD [17,18].

To enhance the accuracy and flexibility of these models, researchers have explored various extensions of classical calculus. One such advancement is fractional calculus, which generalizes the concept of derivatives and integrals to non-integer orders. This naturally leads to the intriguing question: can the notion of derivatives be extended beyond integer orders to include, for example, half-order derivatives? The answer is affirmative, and such extensions are made possible through the framework of fractional calculus [19]. Unlike classical derivatives, fractional derivatives are defined in a non-local manner, meaning they account for the entire history of a function rather than just its instantaneous rate of change. This memory-dependent nature makes them especially powerful for modeling real-world systems where past states influence present behavior. Over the past few years, fractional differential equations have become an essential tool in various research fields, including viscoelasticity [20], electrical engineering [21], diffusion processes [22], biological systems [23], etc. By employing fractional calculus the models offer more precise representations of various complex phenomena. Various types of fractional derivatives are discussed in the literature, each with unique properties and applications. Among them, the Caputo derivative is particularly well-known and widely used. Its popularity stems from its suitability for initial value problems, as it allows for straightforward interpretation when dealing with real-world applications where beginning conditions are described in terms of integer-order derivatives. The Caputo derivative has been widely used in numerous epidemic models, demonstrating its effectiveness. Calatayud *et al*. [24] conducted a study focused on the interpretation of Caputo-type fractional compartmental models. Saeedian *et al*. [25] investigate the effects of memory on epidemic dynamics using the SIR model, demonstrating that the system’s behavior significantly depends on the strength of memory effects, which are determined by the order of the fractional derivatives. Dutta *et al*. [26] examined the spread of infectious diseases using the Caputo derivative, illustrating how public attitudes toward vaccination, social behaviors, and government actions influence epidemic outcomes. The results suggest that implementing control measures not only reduces disease prevalence but also mitigates the economic impact of the epidemic. A Caputo-type SIR epidemic model incorporating an awareness campaign strategy was proposed in [27]. Khan *et al*. [16] explored the impact of vaccination on controlling monkeypox infection using the Caputo derivative. Alaje and Olayiwola [28] investigated a fractional-order COVID-19 model with a focus on vaccine distribution.

Several recent studies have investigated the mathematical model of LSD to better understand its transmission dynamics and control strategies [7,17,29–32]. Elsonbaty *et al*. [7] explored the non-linear dynamics of a discrete fractional model of LSD. In [29] a fractional-order mathematical model of LSD has been investigated using the Caputo approach. Although the latest LSD mathematical models [17,31] investigated only the role of vaccination in LSD dynamics, these models usually do not consider the role of both quarantine and vaccination in the dynamics of the LSD virus. In [32], a mathematical model was introduced to examine the LSD transmission in dairy cows, accounting for both direct and mosquito-borne transmission. The model includes a vaccination component, and the analysis indicated that increasing the vaccination rate reduces the levels of infection. Mani *et al*. [33] investigated an SVEIR-type model for LSD using the Caputo-Fabrizio fractional derivative. Rathee *et al*. [34] developed a Caputo fractional SVEIR model to examine the dynamics of LSD transmission in cattle, employing a predictor–corrector numerical approach.

In our study, we propose an SVEIQR mathematical model for LSD. The novelty of our approach lies in integrating vaccination strategies for susceptible cattle and quarantine measures for the exposed cattle population. The quarantine strategy used in our model is based on the experimental studies [10] and [11]. The primary goal is to construct a reliable framework that facilitates a deeper understanding of disease dynamics. To enhance the accuracy and predictive capability of the model, we employ the Caputo fractional derivative. Fractional calculus possesses a unique ability to depict dynamics that are both non-local and reliant on memory. The choice of the Caputo fractional derivative is motivated by its ability to incorporate local initial conditions into the model formulation. Furthermore, the Caputo derivative of a constant equals zero, preserving consistency with classical differential equations. This methodology accounts for memory effects and long-range dependence, which are often neglected in the integer order model, allowing for a more precise representation of epidemiological dynamics. We aim to improve the precision of the predictions in the LSD model by applying the Caputo fractional derivative. This will result in more efficient control strategies and treatment interventions for LSD.

The document is outlined as follows. Sect 2 presents essential preliminaries regarding fractional derivatives. In Sect 3, focus on the development of the model including non-negativity, boundedness, existence of a unique solution, equilibria, bifurcation, stability and sensitivity. Sect 4 presents the numerical procedure for the solution of the model. Sect 5 focuses on the numerical investigation. Finally, the conclusion is given in Sect 6.

## 2 Preliminaries

In this section, some key formulas that will be applied in formulating the problem are presented [35].

**Definition 2.1.** The fractional integral of order υ>0 of a function *f* is defined as:

$${𝐼}^{\upsilon }f(t)=\frac{1}{\Gamma (\upsilon )}\int_{0}^{t}(t-\xi {)}^{\upsilon -1}f(\xi )d\xi ,$$ (2.1)

where Γ(.) is Gamma function.

**Definition 2.2.** The Caputo fractional derivative of order υ>0 of a function $f\in {𝐶}^{1}([0,\infty ],\mathbb{R})$, defined as:

$${\;}^{𝙲}{𝙳}_{t}^{\upsilon }f(t)=\frac{1}{\Gamma (1-\upsilon )}\int_{0}^{t}(t-\xi {)}^{-\upsilon }f\prime (\xi )d\xi ,\;0<\upsilon <1,\;t>0.$$ (2.2)

**Definition 2.3.** The Laplace transform of the Caputo derivative of order υ is expressed as:

$$ℒ[{\;}^{𝙲}{𝙳}_{t}^{\upsilon }f(t)](s)=s^{\upsilon }ℒ[f(t)]-\sum_{j=0}^{m-1}f^{\left(j\right)}(0)s^{\upsilon -m-1},\;m-1<\upsilon \leq m,$$ (2.3)

where m∈ℕ.

## 3 Lumpy skin disease model

**Fig 1:**
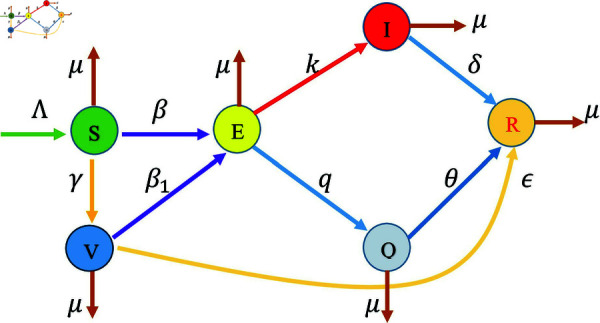
The flow diagram of the LSD model (3.1). The model was inspired by the experimental studies in [10] and [11] which showed that the importance of quarantine and vaccination in mitigating the risk of LSD transmission, and the mathematical modelling studies in [29,17,31] which focused only on the vaccinated effect and did not consider the impact of the quarantine on the LSD infection.

To investigate the quarantine and vaccination roles on the LSD spread, we extend the mathematical model derived in [17], by considering the presence of a quarantine population. We propose an SVEIQR mathematical model for Lumpy skin disease. The total cattle population 𝙽(t) divided into six different classes: 𝚂(t) represents the number of susceptible cattle who are potentially prone to disease through interaction with cattle carrying the virus; those cattle that have been vaccinated are categorized within the vaccinated class 𝚅(t), cattle that have previously been exposed to disease-causing viruses but are not yet infectious are placed in the exposed class 𝙴(t), the cattle that have been identified and confirmed to be positive for LSD infection are represented by 𝙸(t), the cattle in quarantine/isolation class denoted by 𝚀(t). The cattle that have high immunity or have received effective medications that lead to recovery are represented by 𝚁(t). The details about model parameters are given in Table 1. The flow pattern for LSD is illustrated in Fig 1, modelled by the following nonlinear coupled fractional differential equations:

**Table 1:** Parameters description.

Parameters	Description	Values	Source
Λ	Birth rate	4	[17]
μ	Natural death rate	0.2	[17]
β	Infection rate of susceptible cattle	0.039/0.39	[17]
γ	Vaccination rate of susceptible cattle	0.3	[17]
ϵ	Rate at which vaccinated cattle are recovering	0.1	[17]
$\beta_{1}$	Infection rate of vaccinated cattle	0.055	[17]
*k*	Translation rate from 𝙴 to 𝙸	0.59	[17]
*q*	Transfer rate from exposed to quarantined class	0.1886	${\;}^{*}$estimated
δ	Recovering rate of infected cattle	$\frac{1}{7}$	[29]
σ	Disease death rate	0.3	[17]
θ	Recovery rate of quarantined class	0.0348	${\;}^{**}$estimated

Sources denoted by "${\;}^{*}$" and "${\;}^{**}$" relate to parameter values selected within the specified intervals of q∈(1/30,1/4)=(0.03,0.25) [4] and θ∈(1/35,1/28)=(0.028,0.04) [5], respectively.

$${\;}^{𝙲}{𝙳}_{t}^{\upsilon }𝚂(t)=\Lambda -\beta 𝚂𝙸-(\mu +\gamma )𝚂,$$ (3.1a)

$${\;}^{𝙲}{𝙳}_{t}^{\upsilon }𝚅(t)=\gamma 𝚂-\beta_{1}𝚅𝙸-(\epsilon +\mu )𝚅,$$ (3.1b)

$${\;}^{𝙲}{𝙳}_{t}^{\upsilon }𝙴(t)=\beta 𝚂𝙸+\beta_{1}𝚅𝙸-(k+q+\mu )𝙴,$$ (3.1c)

$${\;}^{𝙲}{𝙳}_{t}^{\upsilon }𝙸(t)=k𝙴-(\delta +\sigma +\mu )𝙸,$$ (3.1d)

$${\;}^{𝙲}{𝙳}_{t}^{\upsilon }𝚀(t)=q𝙴-(\theta +\mu )𝚀,$$ (3.1e)

$${\;}^{𝙲}{𝙳}_{t}^{\upsilon }𝚁(t)=\epsilon 𝚅+\delta 𝙸+\theta 𝚀-\mu 𝚁,$$ (3.1f)

with initial conditions


$$𝚂(0)={𝚂}_{0},𝚅(0)={𝚅}_{0},𝙴(0)={𝙴}_{0},𝙸(0)={𝙸}_{0},𝚀(0)={𝚀}_{0},𝚁(0)={𝚁}_{0}.$$

Eqs (3.1a)–(3.1*f*) incorporate the following biological mechanisms as depicted in Fig 1:

- The susceptible cattle are integrated into the population at a rate Λ (see Eq (3.1a)). A constant input of this nature is often considered in LSD disease models (see e.g., [29]). We assume that the rate of new infections resulting from contacts between susceptible and infectious individuals is β𝚂𝙸. The parameter β denotes the infection or transmission rate. This bilinear incidence (i.e., the infection rate per infected individual and susceptible individual is constant), is commonly used in deterministic compartmental models (see [17,29,31]). Susceptible cattle are vaccinated at a rate γ, moving to the vaccinated compartment [17,31]. This represents the implementation of the vaccination program to protect cattle from LSD infection. Finally, we assume all populations have a natural mortality rate μ.
- The vaccinated cattle (see Eq (3.1b)), although protected, can still be exposed to LSD due to vaccine inefficacy or waning immunity, at a rate $\beta_{1}𝚅𝙸$, where $\beta_{1}$ is the infection rate for vaccinated cattle (typically lower than β). Vaccinated cattle can recover at a rate ϵ [17,31].
- The exposed cattle (3.1*c*) progress to the infected compartment at a rate *k*, representing the incubation period after which the cattle become infectious [17,31]. Moreover, it has been experimentally shown in [10] and [11] that the strengthening quarantine and movement control measures can prevent direct LSD transmission. Thus, we assume that the rate of quarantine is represented by *q*.
- The infected cattle (see Eq (3.1d)) can recover from LSD at rate δ. This represents natural recovery or recovery aided by medical intervention. Infected cattle experience disease-induced mortality at a rate σ [17,31].
- The quarantined cattle (see Eq (3.1e)) recover at a rate θ, reflecting recovery during isolation [13]. All other terms have been described above.
- The recovered cattle (see Eq (3.1f)) and all populations have a natural mortality rate μ. All other terms have been described above.

### 3.1 Non-negativity and boundedness

We begin the analysis of model (3.1) by demonstrating its biological feasibility, ensuring that all population variables remain non-negative and bounded [36]. The following results confirm these characteristics.

**Theorem 3.1.** All solutions of the fractional-order model (3.1) that exist in ${\mathbb{R}}_{+}^{6}$ are uniformly bounded and non-negative.

**Proof** The total population is defined as 𝙽(t)=𝚂(t)+𝚅(t)+𝙴(t)+𝙸(t)+𝚀(t)+𝚁(t), then for each μ>0,


$${\;}^{𝙲}{𝙳}_{t}^{\upsilon }𝙽(t)={\;}^{𝙲}{𝙳}_{t}^{\upsilon }𝚂(t)+{\;}^{𝙲}{𝙳}_{t}^{\upsilon }𝚅(t)+{\;}^{𝙲}{𝙳}_{t}^{\upsilon }𝙴(t)+{\;}^{𝙲}{𝙳}_{t}^{\upsilon }𝙸(t)+{\;}^{𝙲}{𝙳}_{t}^{\upsilon }𝚀(t)+{\;}^{𝙲}{𝙳}_{t}^{\upsilon }𝚁(t),\;=\Lambda -\mu (𝚂+𝚅+𝙴+𝙸+𝚀+𝚁)\;=\Lambda -\mu 𝙽$$

Utilizing the standard comparison theorem (SCT) applicable to fractional order [37]

$$𝙽(t)\leq 𝙽(0){𝔼}_{\upsilon }(-\mu t^{\upsilon })+\Lambda t^{\upsilon }{𝔼}_{\upsilon ,\upsilon +1}(-\mu t^{\upsilon }),$$ (3.2)

where ${𝔼}_{q}$ denotes the Mittag-Leffler (ML) function. According to Lemma 5 and Corollary 6 in [38]


$$𝙽(t)\leq \frac{\Lambda }{\mu },\;t⟶\infty .$$

Therefore, all solutions of the fractional order model (3.1) that originate in ${\mathbb{R}}_{+}^{6}$ are confined to the domain Δ, where $\Delta =\{(𝚂,𝚅,𝙴,𝙸,𝚀,𝚁)\in {\mathbb{R}}_{+}^{6}:𝙽(t)\leq \frac{\Lambda }{\mu }\}.$

Now, we are looking for nonnegative solutions for the proposed fractional LSD model (3.1). From Eq (3.1a), we have


$${\;}^{𝙲}{𝙳}_{t}^{\upsilon }𝚂(t)=\Lambda -\beta 𝚂𝙸-(\mu +\gamma )𝚂\;\geq -(\mu +\gamma )𝚂\;\geq -{𝙵}_{1}𝚂,$$

where ${𝙵}_{1}=(\mu \;+\;\gamma ,)$. By SCT for fractional order, using the property of ML function ${𝔼}_{\upsilon ,1}(t)>0,$ for any q∈(0,1) [38]


$$𝚂\geq 𝚂(0){𝔼}_{\upsilon ,1}(-{𝙵}_{1}t^{\upsilon })⟹𝚂\geq 0.$$

From Eq (3.1b), we have


$${\;}^{𝙲}{𝙳}_{t}^{\upsilon }𝚅(t)=\gamma 𝚂-\beta_{1}𝚅𝙸-(\epsilon +\mu )𝚅\;\geq -(\epsilon +\mu )𝚅\;\geq -{𝙵}_{2}𝚅,$$

where ${𝙵}_{2}=\epsilon +\mu$. Therefore,


$$𝚅\geq 𝚅(0){𝔼}_{\upsilon ,1}(-{𝙵}_{2}t^{\upsilon })⟹𝚅\geq 0.$$

From Eq (3.1c), we obtain


$${\;}^{𝙲}{𝙳}_{t}^{\upsilon }𝙴(t)=\beta 𝚂𝙸+\beta_{1}𝚅𝙸-(k+q+\mu )𝙴\;\geq -(k+q+\mu )𝙴\;\geq -{𝙵}_{3}𝙴,$$

where ${𝙵}_{3}=(k+q+\mu )$. Therefore,


$$𝙴\geq 𝙴(0){𝔼}_{\upsilon ,1}(-{𝙵}_{3}t^{\upsilon })⟹𝙴\geq 0.$$

From Eq (3.1d), we obtain


$${\;}^{𝙲}{𝙳}_{t}^{\upsilon }𝙸(t)=k𝙴-(\delta +\sigma +\mu )𝙸\;\geq -(\delta +\sigma +\mu )𝙸\;\geq -{𝙵}_{4}𝙸,$$

where ${𝙵}_{4}=(\delta +\sigma +\mu )$. Therefore,


$$𝙸\geq 𝙸(0){𝔼}_{\upsilon ,1}(-{𝙵}_{4}t^{\upsilon })⟹𝙸\geq 0.$$

From Eq (3.1e), we obtain


$${\;}^{𝙲}{𝙳}_{t}^{\upsilon }𝙴(t)=q𝙴-(\theta +\mu )𝚀\;\geq -(\theta +\mu )𝚀\;\geq -{𝙵}_{5}𝚀,$$

where ${𝙵}_{5}=(\theta +\mu )$. Therefore,


$$𝚀\geq 𝚀(0){𝔼}_{\upsilon ,1}(-{𝙵}_{5}t^{\upsilon })⟹𝚀\geq 0.$$

From Eq 3.1f), we get


$${\;}_{0}^{𝙲}{𝙳}_{t}^{\upsilon }𝚁(t)=\epsilon 𝚅+\delta 𝙸+\theta 𝚀-\mu 𝚁\;\geq -\mu 𝚁\;\geq -{𝙵}_{6}𝚁,$$

where ${𝙵}_{6}=\mu$. Therefore,


$$𝚁\geq 𝚁(0){𝔼}_{\upsilon ,1}(-{𝙵}_{6}t^{\upsilon })⟹𝚁\geq 0.$$

Consequently, the solutions of system (3.1) are demonstrated to be non-negative.

### 3.2 Existence and uniqueness

In this section, we will establish the existence and uniqueness of solutions for the LSD model (3.1).

**Lemma 3.2.** [39] Consider the system

$${\;}_{t_{0}}^{𝙲}{𝙳}_{t}^{\upsilon }x(t)=f(t,x),\;t_{0}>0$$ (3.3)

with primary condition $x(t_{0})=x_{t_{0}}$, where $\upsilon \in (0,1],\;f:[t_{0},\infty ]\times \Omega \to {\mathbb{R}}^{n},\Omega \subseteq R^{n}$, If local Lipschitz condition is fulfilled by *f*(*t*,*x*) for *x*, then there exists a solution of (3.1) on $[t_{0},\infty ]\;\times \;\Omega$ which is unique.

To establish the result, consider the region $\Delta \;\times \;[t_{0},\zeta ]$, where $\Delta =\{(𝚂,𝚅,𝙴,𝙸,𝚀,𝚁)\in {\mathbb{R}}^{6}:\max\{|𝚂|,|𝚅|,|𝙴|,|𝙸|,|𝚀|,|𝚁|\}\leq \Phi \}$ and ζ<+∞. Denote 𝚆=(𝚂,𝚅,𝙴,𝙸,𝚀,𝚁) and $\tilde{W}=(\tilde{𝚂},\tilde{𝚅},\tilde{𝙴},\tilde{𝙸},\tilde{𝚀},\tilde{𝚁})$. Consider a mapping

$𝒦(𝚆)=({𝒦}_{1}(𝚆),{𝒦}_{2}(𝚆),{𝒦}_{3}(𝚆,{𝒦}_{4}(𝚆),{𝒦}_{5}(𝚆),{𝒦}_{6}(𝚆)))$, where


$${𝒦}_{1}(𝚆)=\;\Lambda -\beta 𝚂𝙸-(\mu +\gamma )𝚂,\;{𝒦}_{2}(𝚆)=\;\gamma 𝚂-\beta_{1}𝚅𝙸-(\epsilon +\mu )𝚅,\;{𝒦}_{3}(𝚆)=\;\beta 𝚂𝙸+\beta_{1}𝚅𝙸-(k+q+\mu )𝙴,\;{𝒦}_{4}(𝚆)=\;k𝙴-(\delta +\sigma +\mu )𝙸,\;{𝒦}_{5}(𝚆)=\;q𝙴-(\theta +\mu )𝚀,\;{𝒦}_{6}(𝚆)=\;\epsilon 𝚅+\delta 𝙸+\theta 𝚀-\mu 𝚁.$$

For any $𝚆,\tilde{𝚆}\in \Delta$, we have

$$\begin{array}{cc} \|𝒦(𝚆)-𝒦(\tilde{𝚆})\| & =|{𝒦}_{1}(𝚆)-{𝒦}_{1}(\tilde{𝚆})+|{𝒦}_{2}(𝚆)-{𝒦}_{2}(\tilde{𝚆})+...+|{𝒦}_{6}(𝚆)-{𝒦}_{6}(\tilde{𝚆})|\\ & =|\Lambda -\beta 𝚂𝙸-(\mu +\gamma )𝚂-\Lambda +\beta \tilde{𝚂}\tilde{𝙸}+(\mu +\gamma )\tilde{𝚂}|\\ & \;+|\gamma 𝚂-\beta_{1}𝚅𝙸-(\epsilon +\mu )𝚅-\gamma \tilde{𝚂}+\beta_{1}\tilde{𝚅}\tilde{𝙸}+(\epsilon +\mu )\tilde{𝚅}|\\ & \;+|\beta 𝚂𝙸+\beta_{1}𝚅𝙸-(k+q+\mu )𝙴-\beta \tilde{𝚂}\tilde{𝙸}-\beta_{1}\tilde{𝚅}\tilde{𝙸}+(k+q+\mu )\tilde{𝙴}|\\ & \;+|k𝙴-(\delta +\sigma +\mu )𝙸-k\tilde{𝙴}+(\delta +\sigma +\mu )\tilde{𝙸}|\\ & \;+|q𝙴-(\theta +\mu )𝚀|+|\epsilon 𝚅+\delta 𝙸+\theta 𝚀-\mu 𝚁-\epsilon \tilde{𝚅}-\delta \tilde{𝙸}-\theta \tilde{𝚀}+\mu \tilde{𝚁}|,\\ & \leq 2\beta |𝚂𝙸-\tilde{𝚂}\tilde{𝙸}|+\mu |𝚂-\tilde{𝚂}|+2\gamma |𝚂-\tilde{𝚂}|\\ & \;+2\beta_{1}|𝚅𝙸-\tilde{𝚅}\tilde{𝙸}|+(2\epsilon +\mu )|𝚅-\tilde{𝚅}|\\ & \;+(2k+2q+\mu )|𝙴-\tilde{𝙴}|+(2\delta +\sigma +\mu )|𝙸-\tilde{𝙸}|\\ & \;+(2\theta +\mu )𝚀+\mu |𝚁-\tilde{𝚁}|,\\ & \leq (2\beta_{1}\Phi +\mu )|𝚂-\tilde{𝚂}|+(2\alpha +\mu +2\eta +\gamma_{1})|𝙴-\tilde{𝙴}|\\ & \;+(2\rho +\mu +\delta +\gamma_{2})|{𝙸}_{k}-{\tilde{𝙸}}_{k}|+(2\beta_{1}\Phi +\delta_{1})|{𝙿}_{k}-{\tilde{𝙿}}_{k}|\\ & \;+(\mu +2\omega )|𝚁-\tilde{𝚁}|,\\ & \leq 𝒦\|(𝚂,𝚅,𝙴,𝙸,𝚀,𝚁)-(\tilde{𝚂},\tilde{𝚅},\tilde{𝙴},\tilde{𝙸},\tilde{𝚀},\tilde{𝚁})\|\\ & \leq 𝒦\|𝚆-\tilde{𝚆}\|, \end{array}$$ (3.4)

where $𝒦=\max\{(2\beta_{1}\Phi \;+\;\mu ),(2\alpha \;+\;\mu \;+\;2\eta \;+\;\gamma_{1}),(2\rho \;+\;\mu \;+\;\delta \;+\;\gamma_{2}),(2\beta_{1}\Phi \;+\;\delta_{1}),(\mu \;+\;2\omega )\}.$ Thus, 𝒦(𝚆) meets the Lipschitz condition with respect to 𝚆. Therefore, Lemma 3.2 confirms that there exists a unique solution of fractional-order system (3.1) with initial condition 𝚆(0)=(𝚂(0),𝚅(0),𝙴(0),𝙸(0),𝚀(0),𝚁(0))[40,41].

### 3.3 Basic reproduction ratio and equilibria

**Proposition 1.** *For system (3.1), there exists a positive basic reproduction number* ${ℛ}_{0}$ *such that*

*(i)* *There exists only one equilibrium point* ${ℰ}^{0}$, *when* ${ℛ}_{0}\leq 1$, *and*
*(ii)* *There exist two equilibria,* ${ℰ}^{0}$ *and* ${ℰ}^{*}$, *when* ${ℛ}_{0}>1$.

**Proof**. (i) To find the positive equilibria of the system (3.1), we set the right-hand side to zero. The system demonstrates Lumpy skin disease free equilibrium (LSD-FE) when there are no infected cattle in the community, i.e. 𝙸(t)=0. The LSD-FE equilibrium point is given as ${ℰ}^{0}=({𝚂}_{0},{𝚅}_{0},{𝙴}_{0},{𝙸}_{0},{𝚀}_{0},{𝚁}_{0})=(\frac{\Lambda }{\left(\mu +\gamma \right)},\frac{\Lambda \gamma }{\left(\mu +\epsilon )(\mu +\gamma \right)},0,0,0,\frac{\Lambda \gamma \epsilon }{(\mu +\gamma )(\mu +\epsilon )\mu })$.

The basic reproduction number, commonly denoted by ${ℛ}_{0}$, is used to assess the transmission potential of an infectious disease. It represents the average number of secondary infections caused by a single infected individual introduced into a completely susceptible population [42]. To compute ${ℛ}_{0}$, we apply the next generation matrix approach under the assumption that

$$\frac{dy}{dt}=𝔽(y)-𝕋(y)$$ (3.5)

where $y=(𝙴,𝙸{)}^{\prime }$, 𝔽(y) denotes the influx of new infections and 𝕋(y) signifies the transfer of individuals into and out from the compartments. The Jacobian matrices of 𝔽(y) and 𝕋(y) are examined at the equilibrium point ${ℰ}^{0}$ and are given by

$$𝔽=(\begin{array}{cc} 0 & \;\beta {𝚂}_{0}+\beta_{1}{𝚅}_{0}\\ 0 & \;0 \end{array}),\;𝕋=(\begin{array}{cc} \left(k+q+\mu \right) & 0\\ -k & \left(\delta +\sigma +\mu \right) \end{array}).$$ (3.6)

Then we can calculate $𝔽{𝕋}^{-1}$ as:

$$𝔽{𝕋}^{-1}=(\begin{array}{cc} \frac{(\beta {𝚂}_{0}+\beta_{1}{𝚅}_{0})k}{\left(k+q+\mu )(\delta +\sigma +\mu \right)} & \frac{\beta {𝚂}_{0}+\beta_{1}{𝚅}_{0}}{\left(\delta +\sigma +\mu \right)}\\ 0 & 0 \end{array}).$$ (3.7)

So, ${ℛ}_{0}$ can be obtained by using $\rho (𝔽{𝕋}^{-1})$, where ρ(X) is spectral radius of matrix *X*. Therefore, we have

$${ℛ}_{0}=\frac{[\beta (\mu +\epsilon )+\beta_{1}\gamma ]\Lambda k}{\left(\mu +\epsilon )(\mu +\gamma )(k+q+\mu )(\delta +\sigma +\mu \right)}.$$ (3.8)

(ii) To find the other equilibrium point LSD endemic equilibrium (LSD-EE) ${ℰ}^{*}$, in addition to ${ℰ}^{0}$, let ${ℰ}^{*}=(𝚂*,𝚅*,𝙴*,{𝙸}^{*},{𝚀}^{*},{𝚁}^{*})$ be any equilibrium point of model (3.1) satisfying the equations:

$$\begin{array}{c} 0=\Lambda -\beta {𝚂}^{*}{𝙸}^{*}-(\mu +\gamma ){𝚂}^{*},\\ 0=\gamma {𝚂}^{*}-\beta_{1}{𝚅}^{*}{𝙸}^{*}-(\epsilon +\mu ){𝚅}^{*},\\ 0=\beta 𝚂{𝙸}^{*}+\beta_{1}{𝚅}^{*}{𝙸}^{*}-(k+q+\mu ){𝙴}^{*},\\ 0=k{𝙴}^{*}-(\delta +\sigma +\mu ){𝙸}^{*},\\ 0=q{𝙴}^{*}-(\theta +\mu ){𝚀}^{*},\\ 0=\epsilon {𝚅}^{*}+\delta {𝙸}^{*}+\theta {𝚀}^{*}-\mu {𝚁}^{*}. \end{array}$$ (3.9)

After solving Eq (3.9), we obtain the following expressions for ${𝚂}^{*},{𝚅}^{*},{𝙸}^{*},{𝚀}^{*}$ and ${𝚁}^{*}$ as follows:

$${𝚂}^{*}=\frac{b_{3}b_{4}(\beta_{1}k{𝙴}^{*}+b_{2}b_{4})}{k(k\beta \beta_{1}{𝙴}^{*}+b_{2}b_{4}\beta +b_{4}\beta_{1}\gamma )},\;{𝚅}^{*}=\frac{b_{3}b_{4}^{2}\gamma }{k(k\beta \beta_{1}{𝙴}^{*}+b_{2}b_{4}\beta +b_{4}\beta_{1}\gamma )},\;{𝙸}^{*}=\frac{k{𝙴}^{*}}{b_{4}},\;{𝚀}^{*}=\frac{q{𝙴}^{*}}{b_{5}},\;{𝚁}^{*}=\frac{b_{3}b_{4}^{3}b_{5}\epsilon \gamma +k{𝙴}^{*}(b_{4}q\theta +b_{5}\delta k)(b_{4}(b_{2}\beta +\beta_{1}\gamma )+{𝙴}^{*}k\beta \beta_{1})}{\mu kb_{4}b_{5}(\beta \beta_{1}k{𝙴}^{*}+b_{2}b_{4}\beta +b_{4}\beta_{1}\gamma )},$$ (3.10)

where $b_{1}=\mu +\gamma$, $b_{2}=\epsilon +\mu$, $b_{3}=k+q+\mu$, $b_{4}=\delta +\sigma +\mu$ and $b_{6}=\theta +\mu$. After substituting the values of ${𝚂}^{*},{𝚅}^{*},{𝙸}^{*},{𝚀}^{*}$ and ${𝚁}^{*}$ in the first equation from Eq (3.9), we get the quadratic equation

$$a_{1}{E^{*}}^{2}+a_{2}E^{*}-a_{3}=0,$$ (3.11)

where


$$a_{1}=b_{3}k\beta \beta_{1},$$

$$a_{2}=-(\beta \beta_{1}\Lambda k^{2}+(b_{1}b_{3}\beta_{1}+b_{2}b_{3}\beta )b_{4}k),$$

$$a_{3}=\Lambda b_{4}k(b_{2}\beta +\beta_{1}\gamma )-b_{1}b_{2}b_{3}b_{4}^{2}=b_{1}b_{2}b_{3}b_{4}^{2}({ℛ}_{0}-1).$$

The solutions of Eq (3.11) are given by:


$${E^{*}}^{\pm }=\frac{-a_{2}\pm \sqrt{a_{2}^{2}+4a_{1}a_{3}}}{2a_{2}}.$$

It is clear that *a*_1_>0, therefore if *a*_3_>0 then ${E^{*}}^{+}>0$ and ${E^{*}}^{-}<0$. Let ${𝙴}^{*}={E^{*}}^{+}$, then from Eq (3.10) we obtain:


$${𝚂}^{*}=\frac{b_{3}b_{4}(\beta_{1}k{𝙴}^{*}+b_{2}b_{4})}{k(k\beta \beta_{1}{𝙴}^{*}+b_{2}b_{4}\beta +b_{4}\beta_{1}\gamma )}>0,$$

$${𝚅}^{*}=\frac{b_{3}b_{4}^{2}\gamma }{k(k\beta \beta_{1}{𝙴}^{*}+b_{2}b_{4}\beta +b_{4}\beta_{1}\gamma )}>0,$$

$${𝙸}^{*}=\frac{k{𝙴}^{*}}{b_{4}}>0,$$

$${𝚀}^{*}=\frac{q{𝙴}^{*}}{b_{5}}>0,$$

$${𝚁}^{*}=\frac{b_{3}b_{4}^{3}b_{5}\epsilon \gamma +k{𝙴}^{*}(b_{4}q\theta +b_{5}\delta k)(b_{4}(b_{2}\beta +\beta_{1}\gamma )+{𝙴}^{*}k\beta \beta_{1})}{\mu kb_{4}b_{5}(\beta \beta_{1}k{𝙴}^{*}+b_{2}b_{4}\beta +b_{4}\beta_{1}\gamma )}>0.$$

**Fig 2:**
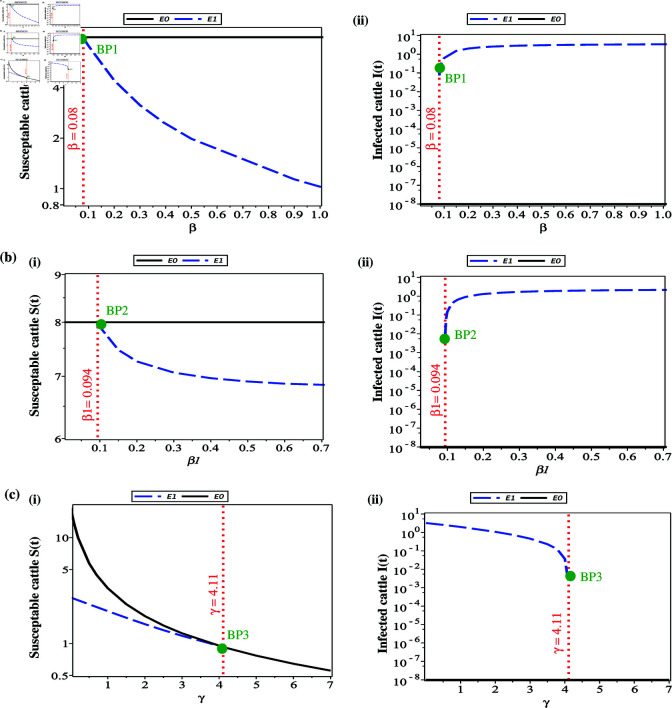
Bifurcation diagram for the two steady states calculated of the model (3.1); ${ℰ}^{0}$ and ${ℰ}^{*}$, as the parameters are varied: (a) rate of infection of susceptible cattle (β), β∈[0.001,1]; (b) rate of infection of vaccinated cattle ($\beta_{1}$), $\beta_{1}\in [0.001,0.7]$; (c) vaccinated rate of of susceptible cattle (γ), γ∈[0.001,7]. Sub-plot (i) shows *S* vs. parameter, while Sub-plot (ii) show *I* vs. parameter. Here "BP1" presents the bifurcation point where the ${ℰ}^{*}$ state bifurcates out of the ${ℰ}^{0}$ state as we increase β above β=0.08, and "BP2" presents the bifurcation point where the ${ℰ}^{*}$ state bifurcates out of the ${ℰ}^{0}$ state as we increase $\beta_{1}$ above $\beta_{1}=0.094$ and "BP3" presents the bifurcation point where the ${ℰ}^{*}$ state bifurcates out of the ${ℰ}^{0}$ state as we decrease γ below γ=4.11.

Therefore, the LSD endemic equilibrium (LSD-EE); ${ℰ}^{*}=({𝚂}^{*},{𝚅}^{*},{𝙴}^{*},{𝙸}^{*},{𝚀}^{*},{𝚁}^{*})$ exists when *a*_3_>0 or ${ℛ}_{0}>1$. In the case of ${ℛ}_{0}>1$, the system’s behavior will be dominated by the endemic endemic equilibrium ${ℰ}^{*}$, which means that the infection persists in the population indefinitely in the long term (the disease becomes endemic). Therefore, intervention is needed to eliminate disease, for instance, increasing γ, θ and ϵ or decreasing β and $\beta_{1}$, which may reduce the ${ℛ}_{0}$ to less than 1, which results in shifting the system toward the disease-free equilibrium. Furthermore, in Fig 2 we show how the two equilibria ${ℰ}^{0}$ and ${ℰ}^{*}$ change as we vary three parameters: (a) rate of infection of susceptible cattle, (b) rate of infection of vaccinated cattle, (c) vaccination rate of susceptible cattle. We can see that an increase in β above β=0.08 or an increase in $\beta_{1}$ above $\beta_{1}=0.094$ or a decrease in γ below γ=4.11 leads to the bifurcation of a ${ℰ}^{*}$ state (which contains LSD infection) from the ${ℰ}^{0}$ state (with no LSD infection).

### 3.4 Stability analysis

In this section, we study the local and global stability of the LSD model (3.1) at its equilibria.

#### 3.4.1 Local asymptotic stability

**Theorem 3.3.** The LSD-FE ${ℰ}^{0}$ is locally asymptotically stable (LAS) if ${ℛ}_{0}<1$ and otherwise unstable.

**Proof.** The linearization of the model (3.1) around the LSD-FE point is given by the Jacobian matrix as:


$$J_{\left(S_{0},V_{0},E_{0},I_{0},Q_{0},R_{0}\right)}=\left[\begin{array}{cccccc} -\gamma -\mu & 0 & 0 & -\frac{\Lambda \beta }{\mu +\gamma } & 0 & 0\\ \gamma & -(\epsilon +\mu ) & 0 & -\frac{\gamma \beta_{1}\Lambda }{\left(\mu +\gamma )(\mu +\epsilon \right)} & 0 & 0\\ 0 & 0 & -(k+q+\mu ) & \frac{\Lambda \beta }{\mu +\gamma }+\frac{\gamma \beta_{1}\Lambda }{\left(\mu +\gamma )(\mu +\epsilon \right)} & 0 & 0\\ 0 & 0 & k & -(\mu +\delta +\sigma ) & 0 & 0\\ 0 & 0 & q & 0 & -\mu -\theta & 0\\ 0 & \epsilon & 0 & \delta & \theta & -\mu \end{array}\right]$$

From the above Jacobian matrix, the four eigenvalues are negative, i.e., −(γ+μ), −(ϵ+μ), − mu , −(μ+θ) and the rest eigenvalues can be obtained by the characteristic equation below:

$$\lambda^{2}+a_{1}\lambda +a_{2}=0$$ (3.12)

Moreover, using the Routh-Hurwitz criteria, Eq (3.12) has negative real parts when


$$a_{1}=k+q+2\mu +\delta +\sigma >0$$

and


$$a_{2}=(k+q+\mu )(\delta +\sigma +\mu )\left(1-\frac{[\beta (\mu +\epsilon )+\beta_{1}\gamma ]\Lambda k}{\left(\mu +\epsilon )(\mu +\gamma )(k+q+\mu )(\delta +\sigma +\mu \right)}\right)$$

$$=(k+q+\mu )(\delta +\sigma +\mu )(1-{ℛ}_{0})>0.$$

Therefore, the LSD-FE ${ℰ}^{0}$ is LAS when ${ℛ}_{0}<1$, otherwise unstable.

#### 3.4.2 Global asymptotic stability.

In this subsection, the global stability at the equilibrium points of the system (3.1) is demonstrated using Lyapunov theory and justified by the following theorems.

**Theorem 3.4.** The LSD-FE ${ℰ}^{0}$ is globally asymptotically stable (GAS) if ${ℛ}_{0}<1$ and otherwise unstable.

**Proof.** We have the Lyapunov function

$$L_{1}=𝒜𝙴+ℬ𝙸,$$ (3.13)

where 𝒜 and ℬ are some positive constants. Applying the Caputo derivative ${\;}^{𝙲}{\text{D}}_{t}^{\upsilon }$ on $L_{1}$, we have

$${\;}^{𝙲}{𝙳}_{t}^{\upsilon }L_{1}(t)=𝒜{\;}^{𝙲}{𝙳}_{t}^{\upsilon }𝙴(t)+ℬ{\;}^{𝙲}{𝙳}_{t}^{\upsilon }𝙸(t).$$ (3.14)

From the system (3.1), we get

$$\begin{array}{cc} {\;}^{𝙲}{𝙳}_{t}^{\upsilon }L_{1} & =𝒜\left[\beta 𝚂𝙸+\beta_{1}𝚅𝙸-(k+q+\mu )𝙴\right]+ℬ\left[k𝙴-(\delta +\sigma +\mu )𝙸\right]\; \end{array}$$ (3.15)

Let 𝒜=k and ℬ=k+q+μ, we get the following

$${\;}^{𝒞}{𝙳}_{t}^{v}L_{1}=\left[k(\beta 𝚂+\beta_{1}𝚅)-(k+q+\mu )(\delta +\sigma +\mu )\right]𝙸$$ (3.16)

$$\leq (k+q+\mu )(\delta +\sigma +\mu ))({ℛ}_{0}-1)𝙸$$

Because all parameters are positive, if ${ℛ}_{0}\leq 1$ then ${\;}^{𝒞}{𝙳}_{t}^{v}L_{1}\leq 0$ for 𝙸>0. We note that the solutions of system (3.1) converge to Δ, the largest invariant subset of $\left\{{\;}^{𝒞}{𝙳}_{t}^{v}L_{1}=0\right\}$. From Eq (3.16) we have ${\;}^{𝒞}{𝙳}_{t}^{v}L_{1}=0$ if and only if 𝙸=0. The set Δ is invariant, and for any element that belongs to Δ satisfies 𝙸=0 then $\dot{𝙸}=0$. We can see from system (3.1) that

$$𝚂\to \frac{\Lambda }{\left(\mu +\gamma \right)},𝚅\to \frac{\Lambda \gamma }{\left(\mu +\epsilon )(\mu +\gamma \right)},𝚀\to 0,𝚁\to \frac{\Lambda \gamma \epsilon }{(\mu +\gamma )(\mu +\epsilon )\mu },$$ (3.17)

as *t* approaches ∞. From LaSalle’s invariance principle [43], ${ℰ}^{0}$ is GAS.

**Theorem 3.5.** The LSD-EE ${ℰ}^{*}$ is globally asymptotically stable (GAS) in Δ, if ${ℛ}_{0}>1$.

*Proof:* To demonstrate the GAS of ${ℰ}^{*}$, we use the same methodology as in [44]. Consider the Lyapunov function as:

$${𝔏}_{2}=\frac{1}{2}(𝚂-{𝚂}^{*}{)}^{2}+\frac{1}{2}(𝚅-{𝚅}^{*}{)}^{2}+...+\frac{1}{2}(𝚁-{𝚁}^{*}{)}^{2}.$$ (3.18)

The differentiation of ${𝔏}_{2}$ with respect to *t*, will give:

$${\;}^{𝙲}{𝙳}_{t}^{\upsilon }{𝔏}_{2}=(𝚂-{𝚂}^{*}){\;}^{𝙲}{𝙳}_{t}^{\upsilon }𝚂+(𝚅-{𝚅}^{*}){\;}^{𝙲}{𝙳}_{t}^{\upsilon }𝚅+...+(𝚁-𝚁{)}^{*}{\;}^{𝙲}{𝙳}_{t}^{\upsilon }𝚁.$$ (3.19)

By considering the following equation:

$${\;}^{𝙲}{𝙳}_{t}^{\upsilon }𝚂=\Lambda -\beta 𝚂𝙸-(\mu +\gamma )𝚂.$$ (3.20)

At the equilibrium point ${ℰ}^{*}$, we get:

$$0=\Lambda -\beta S^{*}I^{*}-(\mu +\gamma )S^{*}.$$ (3.21)

Subtracting Eqs 3.21 from 3.20, we have:

$${\;}^{𝙲}{𝙳}_{t}^{\upsilon }𝚂=-\beta (𝚂𝙸-{𝚂}^{*}{𝙸}^{*})-(\mu +\gamma )(𝚂-{𝚂}^{*}).$$ (3.22)

Now, consider the 1st term of the right hand side of Eq (3.19) and apply Eq (3.22), we have:

$$(𝚂-{𝚂}^{*}){\;}^{𝙲}{𝙳}_{t}^{\upsilon }𝚂=(𝚂-{𝚂}^{*})\left[-\beta (𝚂𝙸-{𝚂}^{*}{𝙸}^{*})-(\mu +\gamma )(𝚂-{𝚂}^{*})\right].$$ (3.23)

Extending the product, ($𝚂𝙸-{𝚂}^{*}{𝙸}^{*}$), gives:

$$𝚂𝙸-{𝚂}^{*}{𝙸}^{*}=(𝚂-{𝚂}^{*})𝙸+{𝚂}^{*}(𝙸-{𝙸}^{*})$$ (3.24)

Therefore,

$$\left(𝚂-{𝚂}^{*})(𝚂𝙸-{𝚂}^{*}{𝙸}^{*})=(𝚂-{𝚂}^{*}{)}^{2}𝙸+{𝚂}^{*}(𝚂-{𝚂}^{*})(𝙸-{𝙸}^{*}\right)$$ (3.25)

Applying Cauchy–Schwarz inequality in the 2nd term of Eq (3.25), we get:

$$\left|(𝚂-{𝚂}^{*})(𝙸-{𝙸}^{*})|\leq |𝚂-{𝚂}^{*}||𝙸-{𝙸}^{*}\right|$$ (3.26)

To simplify the LHS of Eq (3.26), we use Young’s Inequality, we get


$$|(𝚂-{𝚂}^{*})(𝙸-{𝙸}^{*})|\leq \frac{(𝚂-{𝚂}^{*}{)}^{2}}{2\epsilon }+\frac{\epsilon (𝙸-{𝙸}^{*}{)}^{2}}{2},\;\epsilon >0.$$

Multiplying both sides by $\left|{𝚂}^{*}\right|$, we get:

$$|{𝚂}^{*}(𝚂-{𝚂}^{*})(𝙸-{𝙸}^{*})|\leq |{𝚂}^{*}|\left[\frac{(𝚂-{𝚂}^{*}{)}^{2}}{2\epsilon }+\frac{\epsilon (𝙸-{𝙸}^{*}{)}^{2}}{2}\right],\;\epsilon >0.$$ (3.27)

Therefore, from the 2nd term of equation of (33):

$${𝚂}^{*}(𝚂-{𝚂}^{*})(𝙸-{𝙸}^{*})\leq |{𝚂}^{*}(𝚂-{𝚂}^{*})(𝙸-{𝙸}^{*})|\;\leq |{𝚂}^{*}|\left[\frac{(𝚂-{𝚂}^{*}{)}^{2}}{2\epsilon }+\frac{\epsilon (𝙸-{𝙸}^{*}{)}^{2}}{2}\right].$$ (3.28)

So, Eq (3.25) will become:

$$(𝚂-{𝚂}^{*})(𝚂𝙸-{𝚂}^{*}{𝙸}^{*})\leq (𝚂-{𝚂}^{*}{)}^{2}|𝙸|+|{𝚂}^{*}|\left[\frac{(𝚂-{𝚂}^{*}{)}^{2}}{2\epsilon }+\frac{\epsilon (𝙸-{𝙸}^{*}{)}^{2}}{2}\right].$$ (3.29)

Thus, from Eq (3.23) we have:


$$(𝚂-{𝚂}^{*})\left[-\beta (𝚂𝙸-{𝚂}^{*}{𝙸}^{*})-(\mu +\gamma )(𝚂-{𝚂}^{*})\right]=\left[-\beta (𝚂-{𝚂}^{*})(𝚂𝙸-{𝚂}^{*}{𝙸}^{*})-(\mu +\gamma )(𝚂-{𝚂}^{*}{)}^{2}\right].$$

Therefore,

$$(𝚂-{𝚂}^{*}){\;}^{𝙲}{𝙳}_{t}^{\upsilon }𝚂\leq -\beta \left[(𝚂-{𝚂}^{*}{)}^{2}|𝙸|+|{𝚂}^{*}|\left(\frac{(𝚂-{𝚂}^{*}{)}^{2}}{2\epsilon }+\frac{\epsilon (𝙸-{𝙸}^{*}{)}^{2}}{2}\right)\right].$$ (3.30)

This implies that:

$$(𝚂-{𝚂}^{*}){\;}^{𝙲}{𝙳}_{t}^{\upsilon }𝚂\leq -\beta (𝚂-{𝚂}^{*}{)}^{2}|𝙸|-\beta |{𝚂}^{*}|\left(\frac{(𝚂-{𝚂}^{*}{)}^{2}}{2\epsilon }+\frac{\epsilon (𝙸-{𝙸}^{*}{)}^{2}}{2}\right).$$ (3.31)

Hence, the inequality becomes:

$$(𝚂-{𝚂}^{*}){\;}^{𝙲}{𝙳}_{t}^{\upsilon }𝚂\leq -C_{1}(𝚂-{𝚂}^{*}{)}^{2}-C_{2}(𝙸-{𝙸}^{*}{)}^{2},$$ (3.32)

where


$$C_{1}=\beta \left(|𝙸|+\frac{\left|{𝚂}^{*}\right|}{2\epsilon }\right)>0,\;C_{2}=\frac{\beta |{𝚂}^{*}|\epsilon }{2}>0,$$

where *C*_1_ and *C*_2_ are constants which depend on the bounds of ${𝚂}^{*}$ and 𝙸. Similar strategies may be employed to eliminate the cross terms of the remaining terms in Eq (3.19). Consequently, we have demonstrated that ${\;}^{𝙲}{𝙳}_{t}^{\upsilon }{𝔏}_{2}=0$ exclusively at the endemic equilibrium point EEP and ${\;}^{𝙲}{𝙳}_{t}^{\upsilon }{𝔏}_{2}<0$ at other positive solutions. Consequently, employing the LaSalle invariance principle, we have established that the endemic equilibrium point is globally asymptotically stable. ◻

### 3.5 Sensitivity analysis

Sensitivity analysis is essential for identifying optimal strategies for reducing the transmission of infection. Computing sensitivity indices helps analyze the impact of model parameters on ${ℛ}_{0}$. This analysis identifies the most influential parameters for reducing disease transmission, providing critical insights into optimizing intervention measures to control Lumpy skin disease (LSD).

We employ the normalized forward sensitivity index as described in [45] to determine the sensitivity of model parameters. For a given parameter ϱ, the sensitivity index is defined as:

$$\prod_{\varrho }^{{ℛ}_{0}}=\frac{\partial {ℛ}_{0}}{\partial \varrho }\times \frac{\varrho }{{ℛ}_{0}}.$$ (3.33)

**Fig 3:**
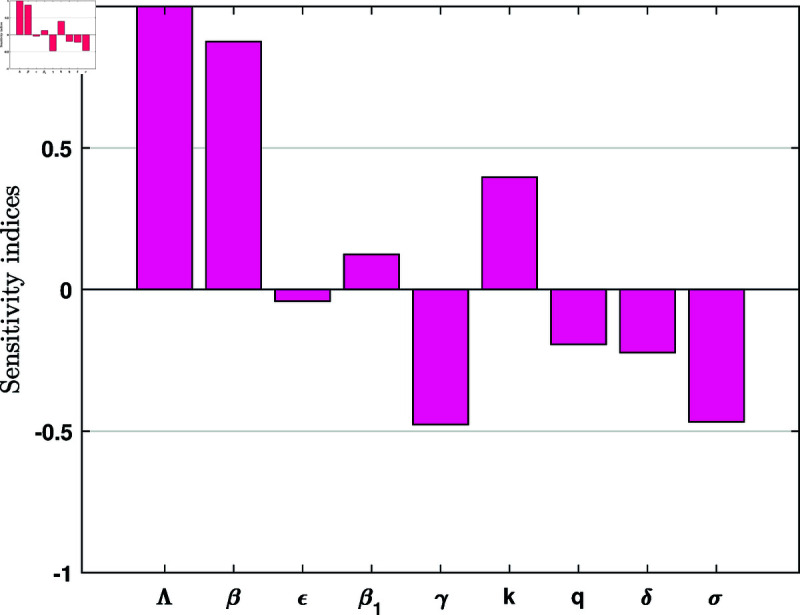
Sensitivity index vs. different parameters.

The sensitivity indices are presented in Table 2 and illustrated in Fig 3.

**Table 2:** Sensitivity index of ${ℛ}_{0}$ to the model parameters.

Parameters	Sensitivity index
Λ	+1.0000
β	+0.8764
ϵ	-0.0412
$\beta_{1}$	+0.1236
γ	-0.4764
*k*	+0.3971
*q*	-0.1927
δ	-0.2222
σ	-0.4667

$$\begin{array}{c} \prod_{\Lambda }^{{ℛ}_{0}}=1>0,\\ \prod_{\beta }^{{ℛ}_{0}}=+\frac{\beta (\mu +\epsilon )}{\beta (\mu +\epsilon )+\beta_{1}\gamma }>0,\\ \prod_{\epsilon }^{{ℛ}_{0}}=-\frac{\beta_{1}\epsilon \gamma }{\left(\mu +\epsilon )(\beta (\mu +\epsilon )+\beta_{1}\gamma \right)}<0,\\ \prod_{\beta_{1}}^{{ℛ}_{0}}=+\frac{\beta_{1}\gamma }{\beta (\mu +\epsilon )+\beta_{1}\gamma }>0,\\ \prod_{\gamma_{1}}^{{ℛ}_{0}}=-\frac{\gamma (\beta (\mu +\epsilon )-\beta_{1}\mu )}{\left(\mu +\gamma )(\beta (\mu +\epsilon )+\beta_{1}\gamma \right)}<0,\\ \prod_{k}^{{ℛ}_{0}}=+\frac{\left(q+\mu \right)}{k+\mu +q}>0,\\ \prod_{q}^{{ℛ}_{0}}=-\frac{q}{\left(k+q+\mu \right)}<0,\\ \prod_{\delta }^{{ℛ}_{0}}=-\frac{\delta }{\left(\delta +\mu +\sigma \right)}<0,\\ \prod_{\sigma }^{{ℛ}_{0}}=-\frac{\sigma }{\left(\delta +\mu +\sigma \right)}<0, \end{array}$$ (3.34)

Identifying the most influential parameters is essential for effectively reducing disease transmission and prevalence. Understanding both the sign and magnitude of their sensitivity indices is key to guiding control strategies. A positive sensitivity index indicates that an increase in the corresponding parameter leads to a rise in the basic reproduction number, ${ℛ}_{0}$, whereas a negative index suggests that increasing the parameter reduces ${ℛ}_{0}$.

The results in Table 2 highlight that parameters such as the recruitment rate (Λ), transmission rate of susceptible cattle (β), vaccination rate (γ), progression rate (*k*), and recovery rate (δ) significantly affect ${ℛ}_{0}$ of the model. Notably, the transmission rate β exhibits a direct proportional relationship with ${ℛ}_{0}$. The sensitivity index $\prod_{\beta }^{{ℛ}_{0}}=+0.8764$ shows that increasing (or decreasing) the transmission rate β by, say, 10%, increases (or decreases) ${ℛ}_{0}$ by 8.764%. This indicates that a higher contact rate between cattle increases the likelihood of infection. As a result, these conditions contribute to establishing an endemic system with a high prevalence of Lumpy skin disease. Reducing β through biosecurity measures, such as controlling insect vectors (e.g., mosquitoes and flies) and limiting cattle movement, is essential for lowering disease prevalence.

Conversely, the vaccination rate γ has the sensitivity index –0.4764, which means that increasing the vaccination rate 10% will decrease ${ℛ}_{0}$ by 4.764%. This highlights the effectiveness of vaccination as a control strategy against Lumpy skin disease, as it reduces the susceptible population of cattle and limits disease transmission. Similarly, the quarantine rate *q* shows a negative sensitivity index, indicating that isolating exposed cattle reduces ${ℛ}_{0}$ by limiting the spread of the virus before cattle become infectious.

Therefore, disease management strategies should focus on parameters with high sensitivity indices, as targeted improvements in these areas can lead to substantial reductions in transmission. For example, strengthening biosecurity practices on cattle farms, implementing timely and widespread vaccination campaigns, enhancing recovery rates through prompt treatment, and restricting the movement of infected animals are all critical control measures. Additionally, ensuring the well-being of livestock and maintaining cleanliness in locations where cattle gather, such as watering sites and shelters, are crucial steps in reducing the spread of Lumpy skin disease.

## 4 Numerical procedure

This section presents a numerical method described in [46,47] for solving the model (3.1). Consider a fractional differential equation of the form

$${\;}^{𝙲}{𝙳}_{t}^{\upsilon }f(t)={𝒢}_{1}(t,f(t)),\;f(0)=f_{0}.$$ (4.1)

Using the fundamental theorem of fractional calculus

$$f(t)=f(0)+\frac{1}{\Gamma (\upsilon )}\int_{0}^{t}(t-\xi {)}^{\upsilon -1}{𝒢}_{1}(\xi ,f(\xi ))d\xi ,$$ (4.2)

at the time instance *t* = *t*_*n* + 1_,*n* = 0,1,2,..., the above equation is reformulated as:

$$\begin{array}{cc} f(t) & =f(0)+\frac{1}{\Gamma (\upsilon )}\int_{0}^{t_{n+1}}{𝒢}_{1}(\xi ,f(\xi )(t_{n+1}-\xi {)}^{\upsilon -1}d\xi ,\\ & =f(0)+\frac{1}{\Gamma (\upsilon )}\sum_{r=0}^{n}\int_{t_{r}}^{t_{r+1}}{𝒢}_{1}(\xi ,f(\xi ))(t_{n+1}-\xi {)}^{\upsilon -1}d\xi . \end{array}$$ (4.3)

Inside the limited range $\left[t_{r},t_{r+1}\right]$ the function ${𝒢}_{1}(\xi ,f(\xi ))$ is approximated using the Lagrangian interpolation method [46,47], we have

$$P_{r}(\xi )≃\frac{{𝒢}_{1}(t_{r},f_{r})}{h}(\xi -t_{r-1})-\frac{{𝒢}_{1}(t_{r-1},f_{r-1})}{h}(\xi -t_{r}).$$ (4.4)

Eq (4.4) can be included in Eq (4.3), and by performing the same steps in [46], we obtain

$$\begin{array}{cc} f(t_{n+1})= & f(0)+\frac{1}{\Gamma (\upsilon )}\sum_{r=0}^{n}(\frac{h^{\upsilon }{𝒢}_{1}(t_{r},f_{r})}{\upsilon (\upsilon +1)}((n+1-r{)}^{\upsilon }(n-r+2+\upsilon )-(n-r{)}^{\upsilon }\\ & \times (n-r+2+2\upsilon ))-\frac{h^{\upsilon }{𝒢}_{1}(t_{r-1},f_{r-1})}{\upsilon (\upsilon +1)}((n+1-r{)}^{\upsilon +1}-(n-r{)}^{\upsilon }(n-r+1+\upsilon ))). \end{array}$$ (4.5)

## 5 Numerical simulations

This section presents the numerical solution of the fractional LSD model (3.1), obtained using the Lagrange polynomial interpolation numerical scheme for the Caputo derivative [46,47]. The simulations are conducted using the parameter values provided in Table 1 and the initial condition as 𝚂(0)=50,𝚅(0)=4,𝙴(0)=3,𝙸(0)=2,𝚀(0)=1,𝚁(0)=0.

**Fig 4:**
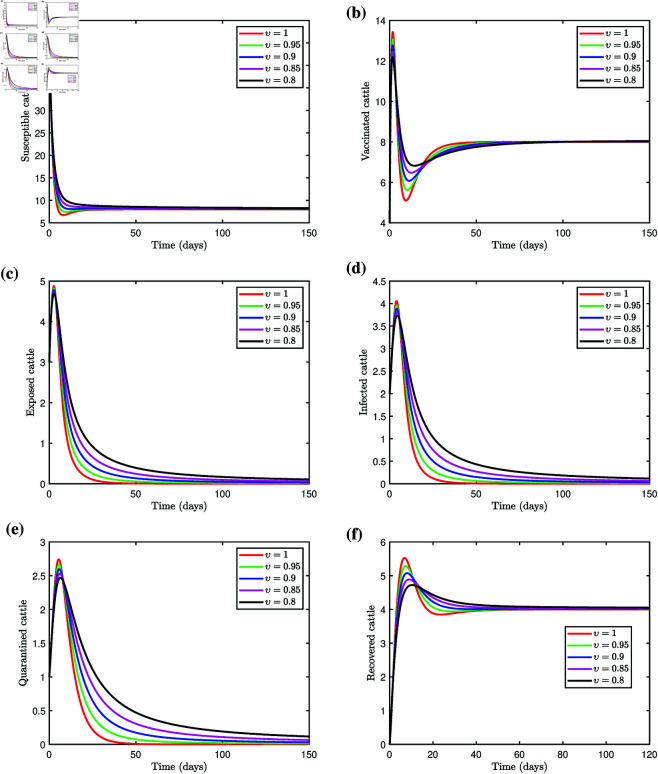
Simulation of the fractional system (3.1) with varying fractional orders, we choose β=0.039 and other parameters as in Table 1, we get ${ℛ}_{0}<1$.

With the transmission rate set to β=0.039 and other parameters as in Table 1, we get ${ℛ}_{0}=0.7053<1$, indicating that the transmission of LSD will eventually decline. This outcome is confirmed through the numerical simulation of the fractional LSD model (3.1), as illustrated in Fig 4a–4f. The simulation shows that the susceptible cattle population declines for different values of the fractional order υ. This highlights the notable influence of υ on the proportion of susceptible individuals. The vaccinated cattle population initially rises due to vaccination of susceptible cattle, then gradually stabilize, with memory effects capturing the impact of historical vaccination efforts. The exposed cattle population spikes early as susceptible and vaccinated cattle contract the virus. For υ=1, where no memory effect is considered, the peak is more prominent, followed by a rapid decline. Introducing the memory effect with υ=0.95,0.9,0.85,0.8 lowers the peak and a more gradual decline over time, which is consistent with observations from real-world epidemics. The solution curve shows that fractional order values influence the proportion of exposed cattle. Infected cattle populations exhibit a similar pattern, where the maximum levels of infection decrease as the fractional order υ value decreases, reflecting the memory effects on behavior. With a stronger memory effect, as observed for υ=0.8, the infection peak diminishes and the decline becomes more gradual. This behavior closely aligns with real-world scenarios, where infections often persist longer because of extended recovery times and the influence of past disease states on current dynamics. A similar pattern was observed in the cattle population in quarantine. The quarantined population reaches its peak and declines faster, reflecting a system with no memory influence. With the memory effect, the peak is lower and takes a longer time to stabilize, reflecting more realistic epidemic dynamics. The fractional order derivative accounts for the cumulative history of exposed cattle entering quarantine, resulting in a slower buildup and a prolonged presence of cattle in quarantine. The recovered cattle population exhibits a steady increase over time, with slight variations in trajectories depending on fractional orders, ultimately converging to the integer order solution. The solution curve takes longer to reach equilibrium for smaller values of fractional orders. However, the higher values of υ lead to quicker convergence to the equilibrium state. Simulations of the fractional LSD model (3.1) reveal that the solution curves in all population classes converge more rapidly to an equilibrium state with higher fractional orders, whereas convergence is slower with lower fractional orders. The incorporation of memory effects via fractional derivatives offers a more accurate representation of disease dynamics within cattle populations.

**Fig 5:**
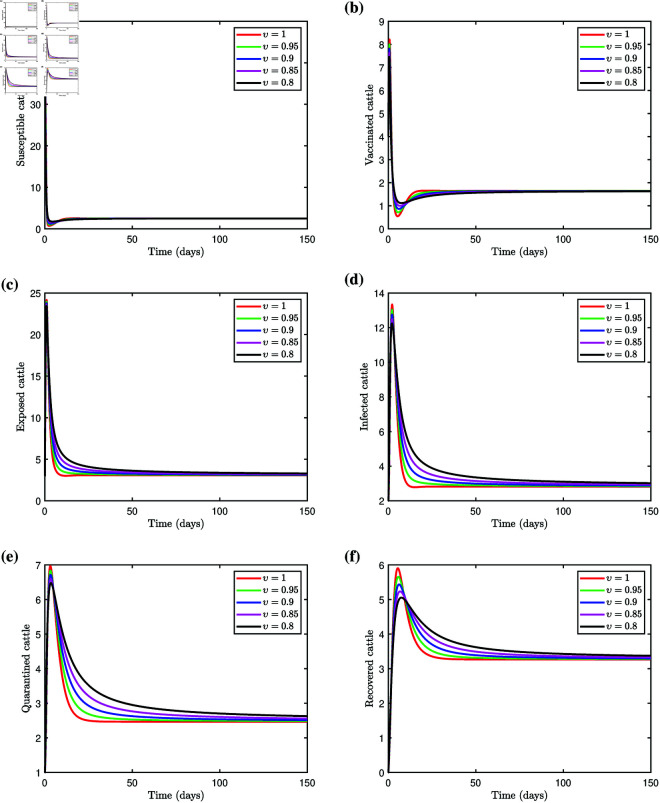
Simulation of the fractional system (3.1) with varying fractional orders, we choose β=0.39 and other parameters as in Table 1, we get ${ℛ}_{0}>1$.

To explore epidemic scenarios, we consider the transmission rate β=0.39. In this case, the ${ℛ}_{0}=3.3387$, which is greater than one and the results are presented in Fig 5a–5f. Visual dynamics shows that the solution curve converges to the EE ${ℰ}^{*}$ for all values of fractional order. The higher fractional orders increase the peak in infected cattle, whereas the peak diminishes as the fractional order decreases. Similar to the case where ${ℛ}_{0}<1$, the results for ${ℛ}_{0}>1$ indicate that the solution curve for all population groups converges to the equilibrium state more quickly at higher fractional orders compared to lower ones.

**Fig 6:**
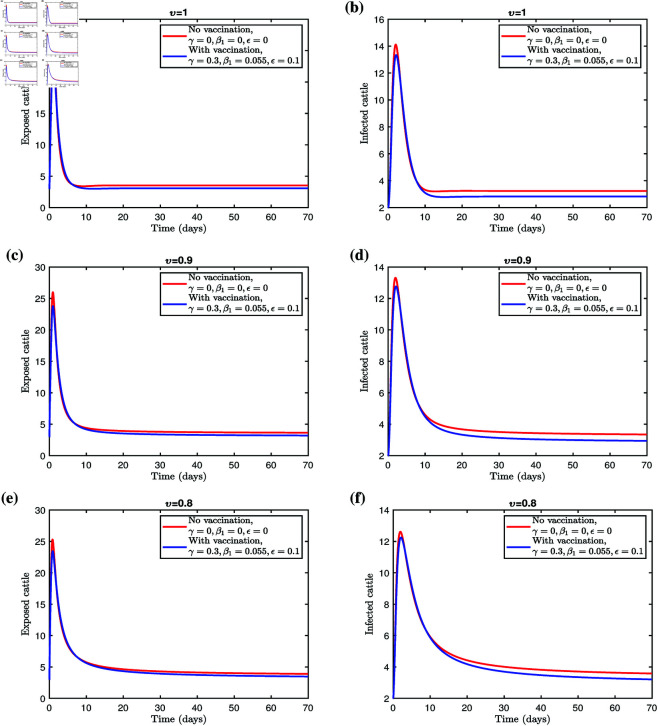
Simulation of the fractional system (3.1) with and without vaccination.

**Fig 7:**
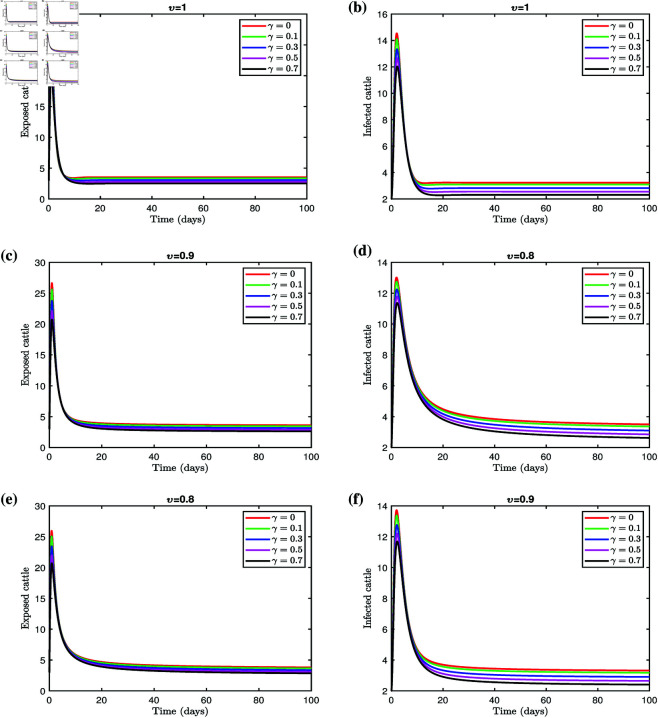
Effect of γ on exposed and infected cattle for different fractional orders.

Figs 6 and 7 illustrate the effectiveness of a vaccination program to vaccinate cattle against LSD. The corresponding results are summarized in Tables 3 and 4, for fractional orders υ=1,0.9, and 0.8. Implementing the vaccination strategy leads to a noticeable reduction in both the exposed and infected populations, highlighting the program’s efficacy. These results underscore the importance of ensuring the availability and effective distribution of vaccines to control infection rates among cattle. The findings suggest that the proposed model is a robust framework for managing and mitigating Lumpy skin disease.

**Table 3:** Impact of vaccination on exposed cattle population at EE point ${ℰ}^{*}$ for different fractional orders.

Vaccination rate	Fractional order		
γ	υ=1	υ=0.9	υ=0.8
0	3.5287	3.6140	3.8108
0.1	3.3719	3.4588	3.6594
0.3	3.0706	3.1610	3.3696
0.5	2.7859	2.8801	3.0974
0.7	2.5178	2.6162	2.8427

**Table 4:** Impact of vaccination on infected cattle at EE point ${ℰ}^{*}$ for different fractional orders.

Vaccination rate	Fractional order		
γ	υ=1	υ=0.9	υ=0.8
0	3.2386	3.3169	3.4998
0.1	3.0947	3.1750	3.3624
0.3	2.8182	2.9025	3.0993
0.5	2.5568	2.6455	2.8523
0.7	2.3108	2.4041	2.6213

**Fig 8:**
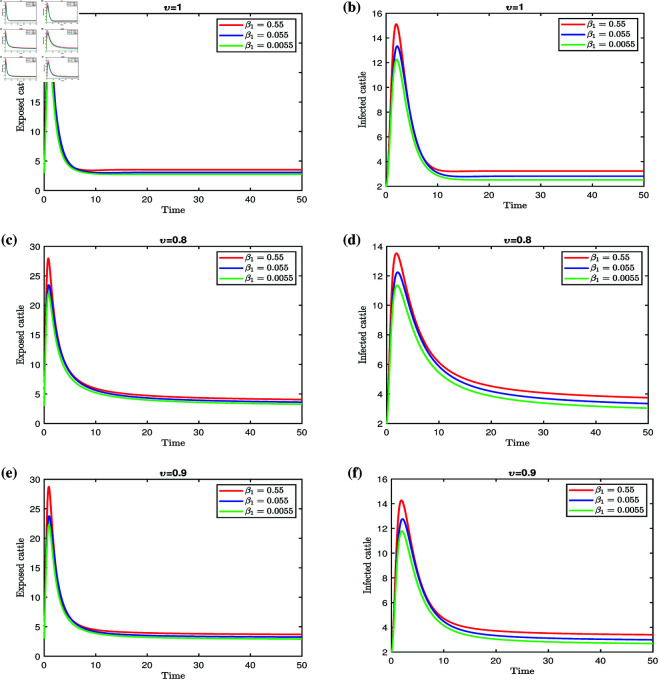
Effect of $\beta_{1}$ on exposed and infected cattle for different fractional orders.

The findings in Fig 8 demonstrate that reducing the contact rate $\beta_{1}$ through targeted strategies can significantly curb epidemic spread. Effective measures to achieve this include implementing targeted prevention initiatives and conducting educational campaigns that emphasize the importance of avoiding contact with insect vectors such as mosquitoes and flies.

**Fig 9:**
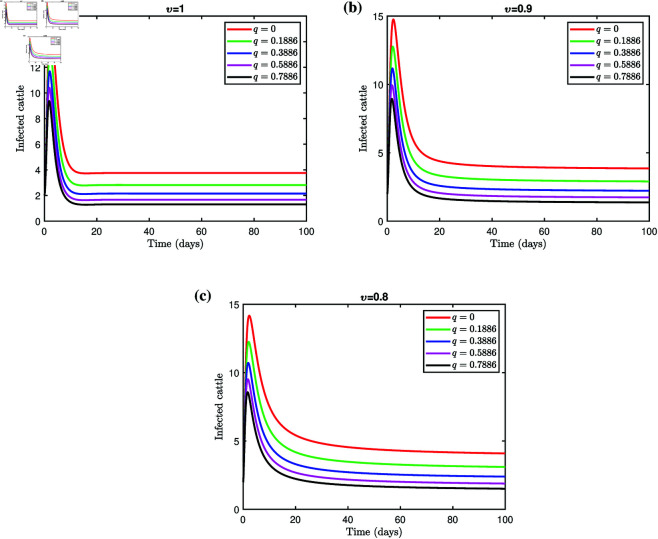
Effect of q on infected cattle populations for different fractional orders.

To prevent the spread of the LSD epidemic among cattle, we examined the effects of different levels of quarantine on LSD dynamics. The visual representation of these dynamics is shown in Fig 9, and the detailed results are presented in Table 5. The findings indicate that disease transmission decreases as the percentage of livestock in quarantine increases. The analysis shows that quarantine can control or eliminate the disease if appropriate quarantine measures are implemented at varying levels, supported by public education.

**Table 5:** Impact of quarantine on infected cattle at EE point ${ℰ}^{*}$ for different fractional orders.

Quarantine rate	Fractional order		
*q*	υ=1	υ=0.9	υ=0.8
0	3.7531	3.8537	4.0890
0.1886	2.8182	2.9025	3.0993
0.3886	2.1479	2.2209	2.3908
0.5886	1.6685	1.7338	1.8852
0.7886	1.3080	1.3682	1.5069

**Fig 10:**
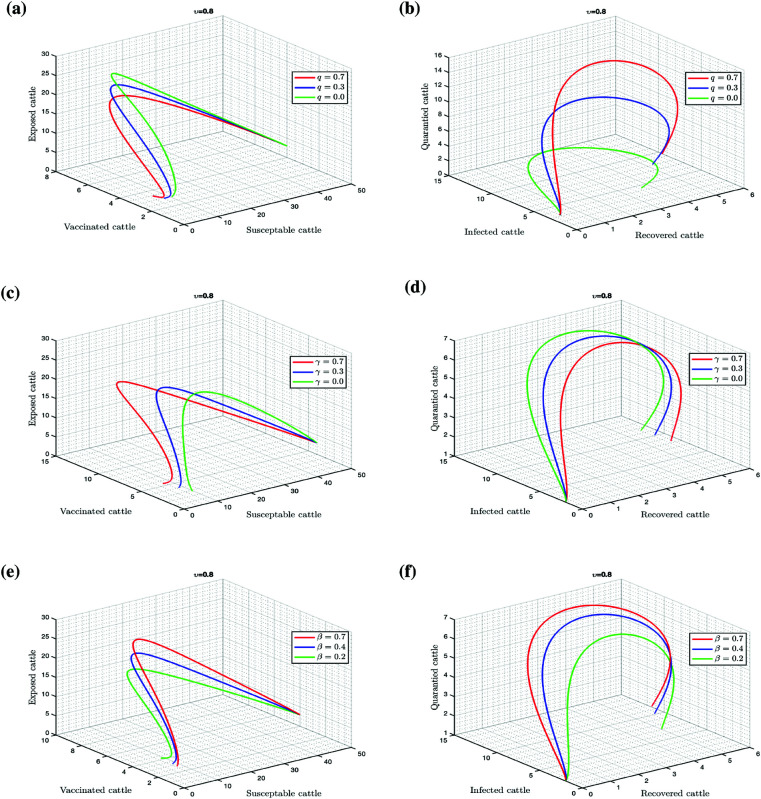
Change in population dynamics of model (3.1), as we vary the following parameters: (a) and (b) quarantine rate q of exposed cattle; (c) and (d) vaccination rate γ of susceptible cattle; (e) and (f) infection rate β of susceptible cattle.

Fig 10 presents the transient dynamics as we change some parameters. In sub-plots (a) and (b), we can see that as *q* increases, the exposed, susceptible, vaccinated and infected cattle decrease, while the recovered population increases. Sub-plots (c) and (d) show that the increase in vaccination rate led to a decrease in the susceptible, exposed and infected populations. The last two sub-plots (e) and (d) demonstrate that increasing the infection rate of susceptible cattle increases the exposed, quarantined, vaccinated and recovered cattle populations.

**Fig 11:**
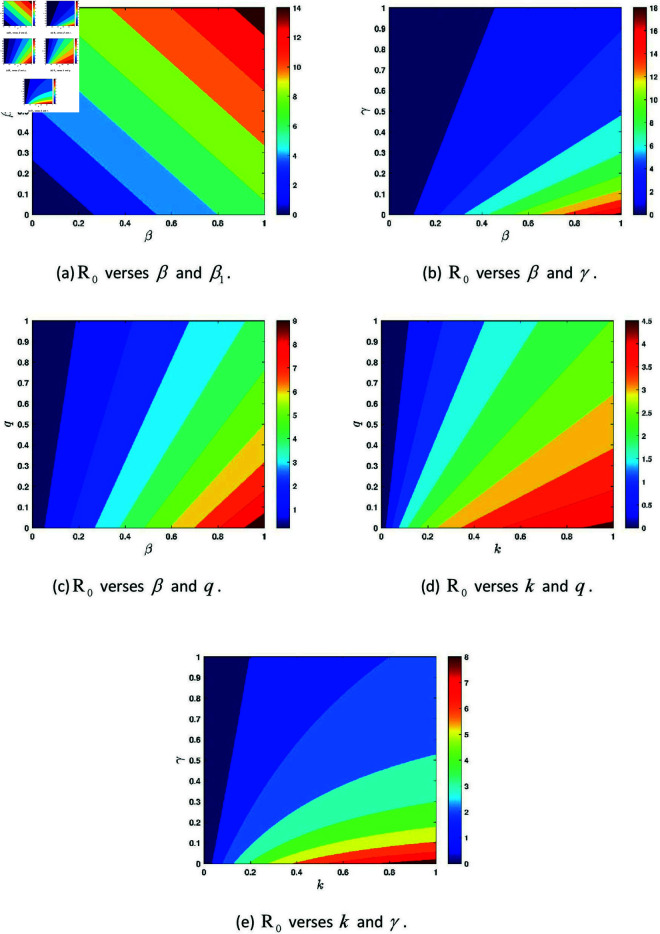
Variations in the basic reproduction number with key parameters of the model (3.1).

The phase planes shown in Fig 11 illustrate the combined effects of several key parameters on the behavior of the basic reproduction number ${ℛ}_{0}$. As observed in Fig 11b, when β and γ vary, the behavior of ${ℛ}_{0}$ changes with smaller ${ℛ}_{0}$ values corresponding to lower β and higher γ. Similarly, we can see that the other parameters influence disease dynamics.

## 6 Conclusion

This study thoroughly investigates the Lumpy skin disease model, incorporating vaccination and quarantine strategies. The Caputo fractional derivative is used to extend the integer-order model to its fractional form. Using an appropriate qualitative method, the existence and uniqueness of solutions for the dynamic model were established. The validity of the model is confirmed by qualitative analysis, such as existence and uniqueness, positivity and boundedness of the solution. The equilibrium points of the model were determined, and stability analysis was conducted for both the disease-free state ℰ and the endemic state ${ℰ}^{*}$. The basic reproduction number was calculated, and the effects of key parameters were analyzed using normalized forward sensitivity indices. A Lagrange polynomial interpolation-based numerical scheme was employed to solve the fractional model, with graphical results presented for varying model parameters and fractional order υ. It is observed that the total number of infections during the disease is influenced by the fractional-order, which represents the memory property. The solution curve takes longer to reach the equilibrium point for lower fractional order values. The impact of vaccination compared to no vaccination was investigated by simulation. The results show that vaccination plays an important role in disease control. Increasing vaccination coverage among cattle reduces infection rates. This finding is consistent with what was achieved in the research [32]. Furthermore, we examined the effect of quarantine measures on the infected cattle population. The results indicate that quarantining exposed cattle is highly effective in curbing the spread of the disease. Compared to the previous studies [7] and [17,18] our model provides a more comprehensive framework by integrating both vaccination and quarantine, with the fractional-order approach enhancing prediction accuracy. Our findings highlight the effectiveness of the strategies adopted in improving the accuracy of the prediction, offering more reliable information on the dynamics of the LSD disease. Furthermore, the results indicate that apply vaccination before infection is more effective than quarantine after infection in reducing the prevalence of LSD diseases, but applying both strategies will better control the disease’s spread. To prevent LSD outbreaks, we recommend the following actionable strategies based on our results: ensuring widespread and timely vaccination of susceptible cattle, early detection and isolation, and implementing biosecurity measures to lower disease transmission (for more details, see, e.g., [10] and [11]). These strategies, grounded in model sensitivity analysis and simulations, provide reliable information to stakeholders, including farmers, veterinarians, and policymakers, to effectively manage LSD. Some limitations of this work are assuming a homogeneous cattle population (no spatial spread), not considering explicitly vector dynamics (e.g., flies/mosquitoes), and ignoring the impact of time delays between the infected population and susceptible/vaccinated population. In addition, we have neglected to evaluate the cost-effectiveness of vaccination compared to quarantine. Some future work can explore the model’s dynamics using fractional operators with nonlocal and nonsingular kernels.

## References

[1] Tuppurainen ESM, Oura CAL. Lumpy skin disease: an emerging threat to Europe, the Middle East and Asia. Transbound Emerg Dis. 2012;59(1):40–8. 21749675 10.1111/j.1865-1682.2011.01242.x

[2] Kayesh ME, Hussan MT, Hashem MA, Eliyas M, Anower AM. Lumpy skin disease virus infection: an emerging threat to cattle health in Bangladesh. Hosts Viruses. 2020;7(4):97.

[3] Sprygin A, et al. Lumpy skin disease: current situation in Russia. Transbound Emerg Dis. 2019;66(2):714–7.

[4] World Organization for Animal Health WOAH. Lumpy Skin Disease – Technical Disease Card. [cited 2026 June]. https://www.woah.org/en/disease/lumpy-skin-disease/

[5] Ratyotha K, Prakobwong S, Piratae S. Lumpy skin disease: a newly emerging disease in Southeast Asia. Vet World. 2022;15(12):2764–71. doi: 10.14202/vetworld.2022.2764-2771 36718323 PMC9880836

[6] Verma N, Agarwal N, Misra L. Review of some diseases of dairy animals and treatment by ethno-veterinary medicines. Advancem Medicinal Plants Res. 2023:9–32.

[7] Elsonbaty A, Alharbi M, El-Mesady A, Adel W. Dynamical analysis of a novel discrete fractional lumpy skin disease model. Partial Diff Equ Appl Math. 2024;9:100604. doi: 10.1016/j.padiff.2023.100604

[8] Kumar N, Chander Y, Kumar R, Khandelwal N, Riyesh T, Chaudhary K, et al. Isolation and characterization of lumpy skin disease virus from cattle in India. PLoS One. 2021;16(1):e0241022. doi: 10.1371/journal.pone.0241022 33428633 PMC7799759

[9] Yadav S, Boora A, Devi P, Nishu, Verma N, Singh I, et al. Isolation and molecular characterization of Lumpy skin disease virus fromcattle and the detection of anti-viral antibodies in buffaloes. Indian J Anim Sci. 2024;94(1):34–8. doi: 10.56093/ijans.v94.i1.138729

[10] Namazi F, Khodakaram Tafti A. Lumpy skin disease, an emerging transboundary viral disease: a review. Vet Med Sci. 2021;7(3):888–96. doi: 10.1002/vms3.434 33522708 PMC8136940

[11] Beard PM. Lumpy skin disease: a direct threat to Europe. Vet Rec. 2016;178(22):557–8. doi: 10.1136/vr.i2800 27235496

[12] Momani S, Chauhan RP, Kumar S, Hadid S. Fractional-order measles infection model with vaccination effects. Fractals. 2023;31(10):2340094.

[13] Ahmad MD, Usman M, Khan A, Imran M. Optimal control analysis of Ebola disease with control strategies of quarantine and vaccination. Infect Dis Poverty. 2016;5(1):72. doi: 10.1186/s40249-016-0161-6 27405359 PMC4942907

[14] Ahmad W, Abbas M. Effect of quarantine on transmission dynamics of Ebola virus epidemic: a mathematical analysis. Eur Phys J Plus. 2021;136(4). doi: 10.1140/epjp/s13360-021-01360-9

[15] Elaiw AM, Almuallem NA, Hobiny A. Effect of RTI drug efficacy on the HIV dynamics with two cocirculating target cells. J Comput Anal Appl. 2017;23(1).

[16] Khan MA, DarAssi MH, Ahmad I, Seyam NM, Alzahrani E. The transmission dynamics of an infectious disease model in fractional derivative with vaccination under real data. Comput Biol Med. 2024;181:109069. 39182370 10.1016/j.compbiomed.2024.109069

[17] Butt AI, Aftab H, Imran M, Ismaeel T. Mathematical study of lumpy skin disease with optimal control analysis through vaccination. Alexandria Eng J. 2023;72:247–59.

[18] Magori-Cohen R, Louzoun Y, Herziger Y, Oron E, Arazi A, Tuppurainen E, et al. Mathematical modelling and evaluation of the different routes of transmission of lumpy skin disease virus. Vet Res. 2012;43(1):1. doi: 10.1186/1297-9716-43-1 22236452 PMC3268087

[19] Ross B. Development of fractional calculus 1695 –1900. Historia Mathematica. 1977;4(1):75–89.

[20] Fang C, Shen X, He K, Yin C, Li S, Chen X, et al. Application of fractional calculus methods to viscoelastic behaviours of solid propellants. Philos Trans A Math Phys Eng Sci. 2020;378(2172):20190291. doi: 10.1098/rsta.2019.0291 32389088

[21] Hristov J. Electrical circuits of non-integer order: Introduction to an emerging interdisciplinary area with examples. Analysis and simulation of electrical and computer systems. Cham: Springer; 2017. p. 251–73.

[22] Sene N. Fractional model for a class of diffusion-reaction equation represented by the fractional-order derivative. Fractal Fraction. 2020;4(2):15.

[23] Magin RL. Fractional calculus models of complex dynamics in biological tissues. Comput Math Appl. 2010;59(5):1586–93.

[24] Calatayud J, Jornet M, Pinto CM. On the interpretation of Caputo fractional compartmental models. Chaos Solitons Fract. 2024;186:115263.

[25] Saeedian M, Khalighi M, Azimi-Tafreshi N, Jafari GR, Ausloos M. Memory effects on epidemic evolution: the susceptible-infected-recovered epidemic model. Phys Rev E. 2017;95(2–1):022409. doi: 10.1103/PhysRevE.95.022409 28297983 PMC7217510

[26] Dutta P, Santra N, Samanta G, De la Sen M. Nonlinear SIRS fractional-order model: analysing the impact of public attitudes towards vaccination, government actions, and social behavior on disease spread. Mathematics. 2024;12(14).

[27] Akdim K, Ez-Zetouni A, Zahid M. The influence of awareness campaigns on the spread of an infectious disease: a qualitative analysis of a fractional epidemic model. Modelling earth systems and environment. 2021:1–9. 10.1007/s40808-021-01158-9 PMC8029611 33851007

[28] Alaje AI, Olayiwola MO. A fractional-order mathematical model for examining the spatiotemporal spread of COVID-19 in the presence of vaccine distribution. Healthc Analyt. 2023;4:100230. doi: 10.1016/j.health.2023.100230

[29] Narwal Y, Rathee S. Fractional order mathematical modeling of lumpy skin disease. Commun Fac Sci Univ Ank Ser A1 Math Stat. 2023;73(1):192–210. doi: 10.31801/cfsuasmas.1207144

[30] Ramaswamy R, Mani G, Mohanraj R, Mohanraj R, Ege O. Mathematical SEIR model of the lumpy skin disease using caputo-fabrizio fractional-order. Eur J Pure Appl Math. 2025;18(2):5933. doi: 10.29020/nybg.ejpam.v18i2.5933

[31] El-Mesady A, Elsadany AA, Mahdy AM, Elsonbaty A. Nonlinear dynamics and optimal control strategies of a novel fractional-order lumpy skin disease model. J Comput Sci. 2024;79:102286.

[32] Falowo OD, Owolabi JA, Oludoun OY, Akingbade R. Mathematical modelling of lumpy skin disease in dairy cow. IOP Conf Ser: Earth Environ Sci. 2023;1219(1):012007. doi: 10.1088/1755-1315/1219/1/012007

[33] Mani G, Gnanaprakasam AJ, Ramalingam S, Omer ASA, Khan I. Mathematical model of the lumpy skin disease using Caputo fractional-order derivative via invariant point technique. Sci Rep. 2025;15(1):9112. doi: 10.1038/s41598-025-92884-y 40097509 PMC11914601

[34] Rathee S, Narwal Y, Bansal K, Emadifar H. Sensitivity analysis of fractional order SVEIR lumpy skin disease model. Alexandria Eng J. 2025;119:609–22.

[35] Kilbas AA, Srivastava HM, Trujillo JJ. Theory and applications of fractional differential equations. Elsevier; 2006.

[36] Belgaid Y, Helal M, Lakmeche A, Venturino E. A mathematical study of a coronavirus model with the caputo fractional-order derivative. Fractal Fract. 2021;5(3):87. doi: 10.3390/fractalfract5030087

[37] Choi SK, Kang B, Koo N. Stability for Caputo fractional differential systems. Abstr Appl Anal. 2014;2014(1):631419.

[38] Huo J, Zhao H, Zhu L. The effect of vaccines on backward bifurcation in a fractional order HIV model. Nonl Anal: Real World Appl. 2015;26:289–305.

[39] Li Y, Chen Y, Podlubny I. Stability of fractional-order nonlinear dynamic systems: Lyapunov direct method and generalized Mittag–Leffler stability. Comput Math Appl. 2010;59(5):1810–21.

[40] Das M, Samanta G, De la Sen M. Stability analysis and optimal control of a fractional order synthetic drugs transmission model. Mathematics. 2021;9(7):703. doi: 10.3390/math9070703

[41] Li H-L, Zhang L, Hu C, Jiang Y-L, Teng Z. Dynamical analysis of a fractional-order predator-prey model incorporating a prey refuge. J Appl Math Comput. 2016;54(1–2):435–49. doi: 10.1007/s12190-016-1017-8

[42] van den Driessche P, Watmough J. Reproduction numbers and sub-threshold endemic equilibria for compartmental models of disease transmission. Math Biosci. 2002;180:29–48. doi: 10.1016/s0025-5564(02)00108-6 12387915

[43] La Salle JP. The stability of dynamical systems. Society for Industrial and Applied Mathematics; 1976.

[44] Goutam S, Pabel S, Abrar F, Amit S. Mathematical modeling of lumpy skin disease: new perspectives and insights. Partial Diff Equ Appl Math. 2025;14:101218.

[45] Chitnis N, Hyman JM, Cushing JM. Determining important parameters in the spread of malaria through the sensitivity analysis of a mathematical model. Bull Math Biol. 2008;70(5):1272–96. doi: 10.1007/s11538-008-9299-0 18293044

[46] Solís-Pérez JE, Gómez-Aguilar JF, Atangana A. A fractional mathematical model of breast cancer competition model. Chaos Solitons Fract. 2019;127:38–54.

[47] Toufik M, Atangana A. New numerical approximation of fractional derivative with non-local and non-singular kernel: application to chaotic models. Eur Phys J Plus. 2017;132(10). doi: 10.1140/epjp/i2017-11717-0

